# Lysine acetylation modulates s‐OPA1 GTPase activity and oligomerization in mitochondrial membrane remodeling

**DOI:** 10.1002/pro.70179

**Published:** 2025-05-29

**Authors:** Javaid Jabbar, Bakht Afroze, Naomi X. Y. Ling, Jonathan S. Oakhill, Isabelle Rouiller

**Affiliations:** ^1^ Department of Biochemistry & Pharmacology Bio21 Molecular Science and Biotechnology Institute, University of Melbourne Parkville Victoria Australia; ^2^ ARC Centre for Cryo‐electron Microscopy of Membrane Proteins Parkville Victoria Australia; ^3^ St. Vincent's Institute of Medical Research Fitzroy Victoria Australia; ^4^ Department of Medicine University of Melbourne Parkville Victoria Australia; ^5^ Faculty of Health Sciences Australian Catholic University Melbourne Victoria Australia

**Keywords:** acetylation, GTPase activity, membrane remodeling, oligomeric assembly, OPA1

## Abstract

Mitochondrial dynamics are regulated by coordinated fission and fusion events that rely on key proteins and lipids organized spatially within the mitochondria. The dynamin‐related GTPase Optic Atrophy 1 (OPA1) is essential for inner mitochondrial membrane fusion and cristae structure maintenance. While post‐translational modifications, particularly lysine acetylation, are emerging as critical regulators of mitochondrial protein function, their impact on OPA1 remains poorly characterized. In this study, we explored the effects of lysine acetylation on the short form of OPA1 (s‐OPA1) using acetylation and deacetylation mimetic mutations. Through a combination of in silico analyses and functional assays, we identified lysine residues in s‐OPA1 that are conserved across species and significantly influence protein stability, GTPase activity, and oligomeric assembly upon acetylation or deacetylation. Our findings reveal that acetylation at K328 and deacetylation at K342 within the G domain enhance the GTPase activity of s‐OPA1 upon lipid membrane binding, whereas deacetylation at K772 abolishes membrane binding‐induced GTPase activity. Negative‐stain transmission electron microscopy indicated that while lysine acetylation does not alter the ability of s‐OPA1 to bind and tubulate liposomes, it significantly impacts higher‐order filament formation. These findings provide novel insights into how acetylation modulates s‐OPA1 function, highlighting a potential mechanism for post‐translational regulation of mitochondrial dynamics. Our study contributes to the understanding of how molecular changes influence broader cellular processes, with implications for mitochondrial function and related disorders.

## 1 INTRODUCTION

Mitochondrial dynamics, encompassing fission and fusion processes, are crucial for maintaining cell health and energy production. These dynamic changes are critical for maintaining mitochondrial integrity, which is vital for metabolic regulation, apoptosis, synthesis of metabolites, production of reactive oxygen species, calcium homeostasis, and regulating immune response (Chen et al. 2023; Tilokani et al. 2018). A key player in this process is Optic Atrophy 1 (OPA1), a dynamin‐related GTPase responsible for the fusion of the inner mitochondrial membrane (Belenguer and Pellegrini 2013). OPA1 also contributes to maintaining mitochondrial DNA copy number, cristae shape, mitophagy, substrate transport, oxidative phosphorylation, and ATP production, apoptosis inhibition, and calcium buffering (Belenguer and Pellegrini 2013; Del Dotto et al. 2018; MacVicar and Langer 2016; Yu‐Wai‐Man et al. 2016). Disruptions in OPA1 function have been implicated in various diseases, including cardiomyopathy (Chen et al. 2012), Behr Syndrome (Bonneau et al. 2014), autosomal dominant optic atrophy (DOA) (Alexander et al. 2000; Delettre et al. 2000), metabolic stroke (Zerem et al. 2019), syndromic parkinsonism, and dementia (Carelli et al. 2015).

In humans, OPA1 exists as eight isoforms that enter the mitochondrial intermembrane space via a mitochondrial‐targeting sequence (MTS) (Del Dotto et al. 2017). After MTS removal, the long form (L‐OPA1) is anchored to the inner membrane. All isoforms have an S1 cleavage site for the OMA1 protease, activated by membrane depolarization, producing the short form (s‐OPA1) (Del Dotto et al. 2018). Four isoforms also feature an S2 cleavage site for YME1L (Anand et al. 2014), while one has an S3 site (Wang et al. 2021). Both L‐OPA1 and s‐OPA1 form oligomers, crucial for remodeling mitochondrial membranes and maintaining their network (Anand et al. 2014).

Aberrant lysine acetylation has been identified as a significant factor associated with late‐onset neurodegenerative diseases (Cohen et al. 2015; Mattson 2003; Min et al. 2010). This post‐translational modification regulates various neuronal proteins, including histones and tubulin, and is crucial for the development, stability, and plasticity of neuronal networks (Mattson 2003). One notable example is the tau protein, where acetylation prevents the degradation of phosphorylated tau (p‐tau) (Min et al. 2010). Recent findings also highlight lysine acetylation as a significant factor influencing TDP‐43 function and aggregation. This modification impairs TDP‐43's ability to bind RNA and leads to the accumulation of insoluble, hyper‐phosphorylated forms of the protein, which resemble the pathological inclusions found in conditions like amyotrophic lateral sclerosis (ALS) and frontotemporal lobar degeneration with TDP‐43 inclusions (FTLD‐TDP) (Cohen et al. 2015). Unlike nuclear and cytoplasmic acetylation mediated by specific lysine acetyltransferases (KATs) (Ali et al. 2018; Drazic et al. 2016), mitochondrial acetylation occurs non‐enzymatically when acetyl‐CoA levels are elevated, such as during feeding (Ali et al. 2018; Anderson and Hirschey 2012; Baeza et al. 2015; Baeza et al. 2016; Choudhary et al. 2014; Paik et al. 1970; Wagner and Hirschey 2014; Weinert et al. 2014).

Despite OPA1's association with various diseases (Alexander et al. 2000; Bonneau et al. 2014; Carelli et al. 2015; Delettre et al. 2000; Zerem et al. 2019), the effects of acetylation on its function and mitochondrial dynamics remain unclear. A previous study has reported OPA1 to be acetylated at K926 and 931 and showed OPA1 acetylation causes a decrease in GTPase activity (Samant et al. 2014). Another study demonstrated that OPA1 acetylation results in mitochondrial fission and pyroptosis in hair cells, causing age‐related hearing loss (Zhang et al. 2024). In both studies (Samant et al. 2014; Zhang et al. 2024), OPA1 was hyperacetylated non‐specifically therefore, the role of site‐specific acetylation on OPA1 function remains unknown.

In this study, we employed bioinformatics to systematically assess the potential impact of acetylation and deacetylation using mimetic mutations (Q for acetylation and R for deacetylation), at 16 sites in OPA1, identifying five sites that may influence its function. We then investigated how mutations at these sites affect OPA1's ability to oligomerize on liposomes mimicking the inner mitochondrial membrane (IMM) and to hydrolyze GTP.

Our results indicated that lysine acetylation or deacetylation did not impair OPA1's ability to bind to lipids but influenced membrane remodeling in a position‐dependent manner. In the apo state, K328Q and K342Q/R mutants formed wider filaments. Similarly, in the pre‐hydrolysis GTP‐bound state, K342Q/R, K579Q, and K772R mutants exhibited increased filament width. Although we observed no correlation between filament width and GTP hydrolysis, specific mutations significantly affected GTPase activity: the acetylation‐mimetic mutation at K328 and the deacetylation‐mimetic mutation at K342 significantly enhanced GTPase activity, whereas the deacetylation‐mimetic mutation at K772 significantly reduced it. Taken together, we propose that lysine acetylation induces conformational changes within the OPA1 dimer influencing its oligomerization and filament formation. Consequently, lysine acetylation or deacetylation at sites that modulate GTPase activity also impacts membrane disassembly dynamics. Proteins with higher GTP hydrolysis activity are likely to disassemble more rapidly from the membrane, while those with lower activity may remain membrane‐bound for extended periods.

## 2 RESULTS

### 2.1 Acetylation mimetic mutations alter protein stability

**FIGURE 1:**
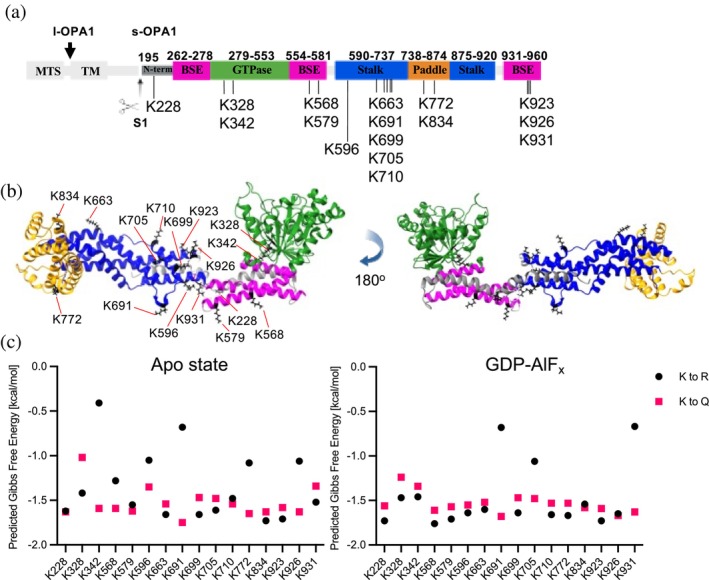
Effects of site‐specific acetylation of s‐OPA1 using acetylation mimetic mutants. (a) Domain model of human OPA1 isoform 1, showing mitochondrial targeting signal (MTS) and transmembrane domain (TM) in white, G‐domain (GTPase) in green, bundle signaling element (BSE) in pink, stalk domain in blue, paddle domain in orange, and N‐terminus in dark gray. L‐OPA1 undergoes proteolytic cleavage at the S1 site at position 195 (indicated by a black arrow), producing s‐OPA1. Residue numbers at the top denote domain boundaries, while known acetylated lysine residues are indicated by the letter “K” followed by the residue number at the bottom. (b) Model of s‐OPA1 in the apo‐state, with domains colored as in panel (a). Acetylated lysine residues are labeled in black and shown as sticks. PDB ID for the model is 8EF7. (c) Predicted changes in Gibbs free energy (Δ*G*, kcal mol^−1^) for s‐OPA1 upon mutation of lysine residues to arginine (black dots) and glutamine (pink squares) at specific positions in the apo‐state (left) and GDP‐ALF_x_‐bound state (right). PDB IDs for the models used are 8EF7 (apo‐state) and 8EEW (GDP‐ALF_x_‐bound state) (Nyenhuis et al. 2023).

Biochemical and functional studies have identified 16 lysine residues in OPA1 that undergo acetylation (Figure 1a). These residues include K228, 328, 342, 568, 579, 596, 663, 691, 699, 705, 710, 772, 834, 923, 926, and 931 (Hornbeck et al. 2015). These acetylation sites span different domains of s‐OPA1, specifically the N terminus helix, the bundle signaling elements (BSE), the G‐domain (GTPase), the stalk domain, and the lipid‐binding paddle domain (Figure 1a,b). Although these acetylation sites in OPA1 were identified in three different species: *Homo sapiens*, *Mus musculus*, *Rattus norvegicus*, the OPA1 sequence is 95.73% conserved in these species, including the lysine residues undergoing acetylation (Figure S1, Supporting Information). Notably, four of the identified acetylation sites are associated with disease‐causing mutations linked to DOA. These mutations are the amino acid substitutions K328R and K342E in the G‐domain and frameshift mutations K663 (resulting in K663N) and K699 (resulting in K699N) (Nyenhuis et al. 2023). These mutations highlight the potential significance of these lysine residues in OPA1 function and their role in the pathogenesis of DOA.

We first calculated the predicted Gibbs free energy (ΔΔ*G*) using DynaMut2 (Rodrigues et al. 2021) to assess if site‐specific acetylation would affect the stability of OPA1 dimer, the building block of s‐OPA1 oligomeric assembly (Nyenhuis et al. 2023; von der Malsburg et al. 2023; Zhang et al. 2020). This analysis was performed for both the apo state (PDB: 8EF7) and the GDP‐Pi analog‐bound state (GDP‐AlF_x_, PDB: 8EEW) (Nyenhuis et al. 2023) (Figure 1c). DynaMut2 revealed position‐specific differences in the predicted Gibbs free energy (ΔΔ*G*) between the apo state and the GDP‐AlF_x_‐bound state (Figure 1c). In the apo state, K228, K579, and K710 showed no difference in ΔΔ*G* when mutated to Q or R. Other lysine residues, including K328, K663, K699, K705, K834, K923, and K931, exhibited increased protein stability when mutated to Q, as the ΔΔ*G* was greater than when mutated to R. Conversely, mutation of K to R at positions 342, 568, 596, 691, 772, and 926 showed increased protein stability compared to mutation to Q, as ΔΔ*G* increased with the R mutation. In the GDP‐AlF_x_‐bound state, most residues, including K228, K328, K342, K568, K579, K596, K663, K699, K710, K772, and K923, showed increased protein stability upon mutation to Q. However, lysine at positions K691, K705, and K931 displayed increased stability upon mutation to R. No significant differences in ΔΔ*G* were observed for K834 and K926 when mutated to Q or R (Figure 1c). Notably, K691 showed a significant increase in protein stability upon mutation to R compared to Q in both states; its location in a flexible loop region (Figure 1b) may explain the drastic difference. The change in ΔΔ*G* between the apo state and the GDP‐AlF_x_‐bound state can be attributed to the conformational change that occurs upon nucleotide binding, leading to the rearrangement of interatomic bonds.

Next, we examined the published structures (PDB: 8EF7 and 8EEW) for interatomic bonds between lysine residues that get acetylated and nearby residues. Out of 16 lysine residues, only 5 showed significant interatomic bonding (Figure S2). In the apo state, in chain A, K328 and K342 formed hydrogen bonds with D273 and D277 in BSE1, respectively. Similarly, K579 in chain B formed bonds with P583 and Y582. In the middle domain, K663 in chain B formed a hydrogen bond with E706 in chain A, and K772 formed a hydrogen bond with D770 in chain A (Figure S2a,b). Upon nucleotide binding (GDP‐AlF_x_), the hydrogen bonding patterns shifted. K328 in both chains continued to form a bond with D273, while K342 in chain B formed a bond with D343. K663 in chain A formed a hydrogen bond with E706 in chain B. No interatomic bonds were observed for K579 and K772 in either chain upon nucleotide binding (Figure S2a,c). Based on our analysis, we selected five positions for in vitro study: K328, K342, K579, K663, and K772, of which K328, K342, and K663 are associated with DOA.

### 2.2 Purification and characterization of s‐OPA1 WT and mimetic mutants

**FIGURE 2:**
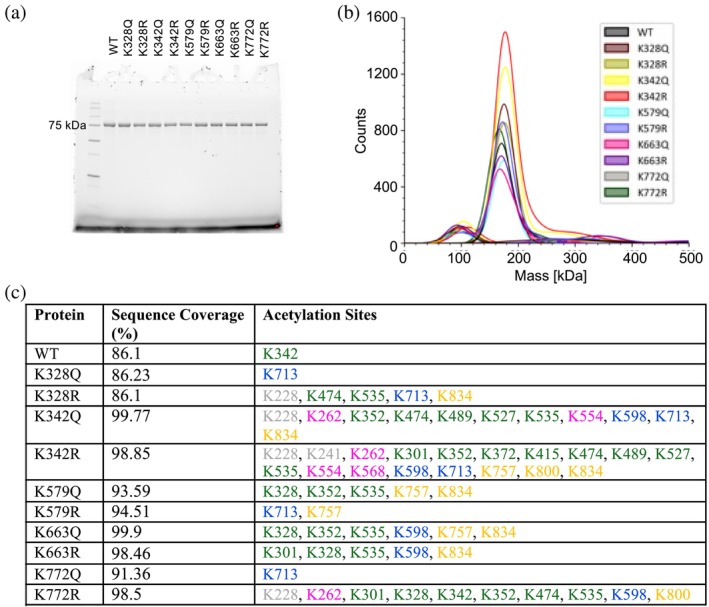
Purification and characterization of s‐OPA1 WT and mimetic mutants. (a) SDS‐PAGE of OPA1 WT and mimetic mutants after size‐exclusion chromatography. (b) Mass photometry profile of s‐OPA1 WT and mutants showing all the proteins are predominantly dimeric. (c) Table showing the results of in gel trypsin digestion followed by mass spectrometry analysis. The lysine residues that were found to be acetylated are colored according to their domain localization in Figure 1a: G‐domain (GTPase) in green, bundle signaling element (BSE) in pink, stalk domain in blue, paddle domain in orange, and N‐terminus in dark gray.

We expressed s‐OPA1 wild‐type (WT) and acetylation mimetic mutants (Q and R) at lysine residues K328, K342, K579, K663, and K772 in *Escherichia coli* BL21 (DE3) cells. All proteins were homogeneously purified and found to exist as dimers in solution by mass photometry, with molecular weights of approximately 167–173 kDa (Figure 2a,b). To validate the identity of the proteins and assess endogenous lysine acetylation potentially acquired during expression, we performed in‐gel trypsin digestion followed by mass spectrometry analysis. Our findings revealed variability in the number of acetylated lysine residues across different proteins. While WT, K328Q, and K772Q displayed a single acetylation site, other mutants showed varying degrees of acetylation, with K342R exhibiting the highest number at 18 sites (Figure 2c).

Among the previously reported acetylation sites (Figure 1a,b), we detected acetylation at K228, K328, K342, K568, and K834 across different mutants (Figure 2c). In addition, we identified several novel acetylation sites including K241, K262, K301, K352, K372, K415, K474, K489, K527, K535, K554, K598, K713, K757, and K800, which have not been reported before. Notably, the acetylation profiles differed between the Q and R mimetic mutants at each site, suggesting that specific mutations may influence the extent and pattern of non‐enzymatic acetylation (Figure 2c). This diversity in lysine acetylation supports a model in which OPA1 is predominantly acetylated through a non‐enzymatic mechanism, likely driven by the high intracellular concentration of acetyl‐CoA in *E. coli* expression systems (Novak et al. 2018).

### 2.3 Lysine acetylation affects s‐OPA1‐mediated membrane remodeling and oligomerization

**FIGURE 3:**
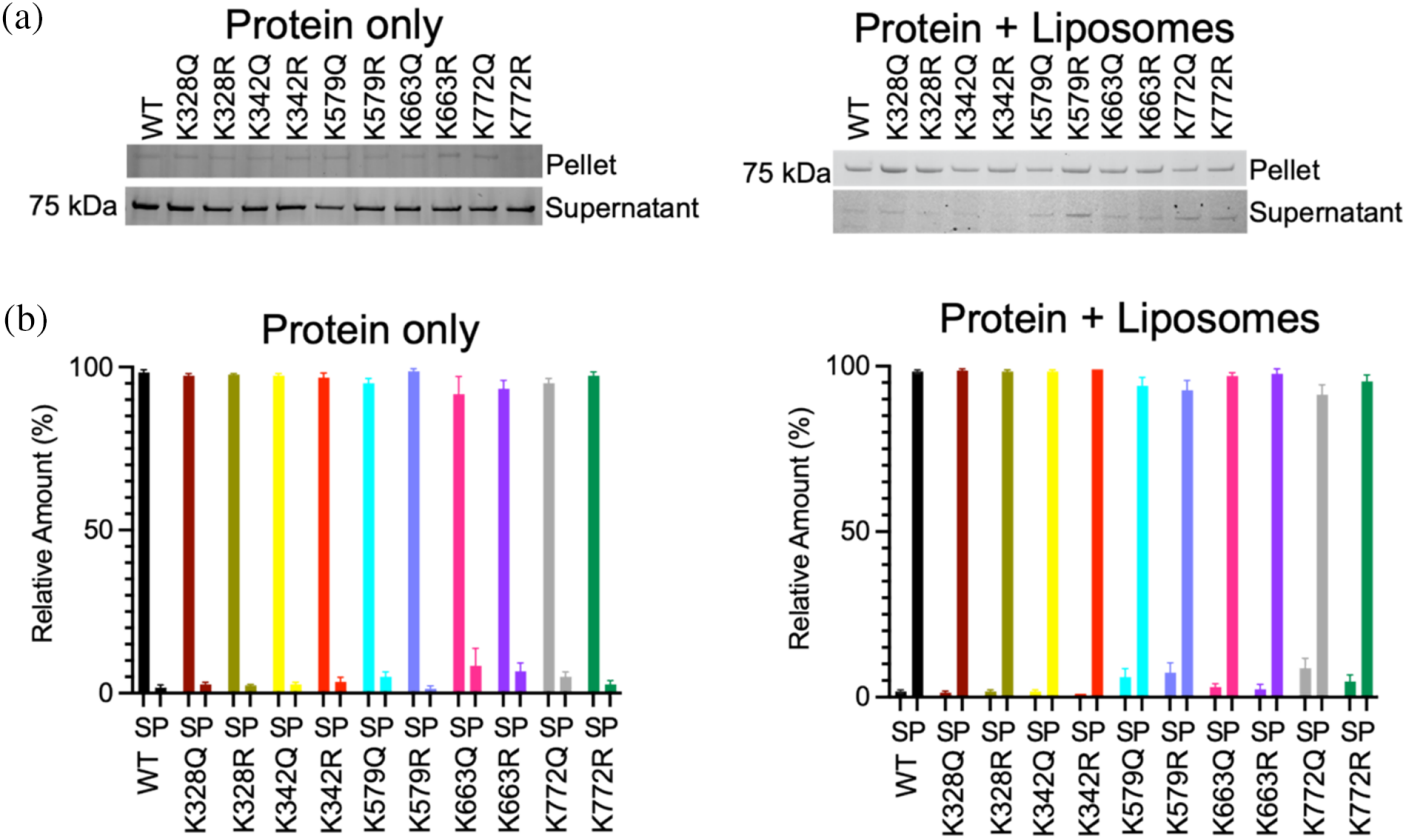
Liposome co‐sedimentation assay of s‐OPA1 WT and acetylation mimetic mutants. (a) Representative SDS‐PAGE gels showing the sedimentation of s‐OPA1 WT and mutants in the absence (left) and presence (right) of cardiolipin‐enriched liposomes. The pellet fraction (top) and supernatant (bottom) are shown. Experiments were conducted in triplicate. (b) Quantification of SDS‐PAGE gels, depicting the relative amount (%) of s‐OPA1 WT and mutants in the pellet and supernatant fractions without liposomes (left) and with liposomes (right). Data represent the mean ± SEM of triplicate experiments.

We performed a liposome co‐sedimentation assay to evaluate whether lysine acetylation affects s‐OPA1 binding to liposomes mimicking the composition of IMM (1‐palmitoyl‐2‐oleoyl‐sn‐glycero‐3‐phosphocholine (POPC), 1‐palmitoyl‐2‐oleoyl‐sn‐glycero‐3‐phosphoethanolamine (POPE), L‐α‐lysophosphatidylinositol (PI), and cardiolipin in a 45:22:8:25 molar ratio) (Chan 2006). SDS‐PAGE analysis of pellet and supernatant fractions showed that both WT and mimetic mutants were present in the pellet in the presence of liposomes, indicating binding. In the absence of liposomes, all proteins remained in the supernatant (Figures 3a and S3). Quantification revealed no significant differences in liposome binding efficiency between WT and mimetic mutants (Figure 3b), suggesting that acetylation or deacetylation at the examined lysine residues does not affect s‐OPA1's ability to associate with IMM‐like liposomes. Additional endogenous lysine acetylation (Figure 2c) did not alter liposome binding.

**FIGURE 4:**
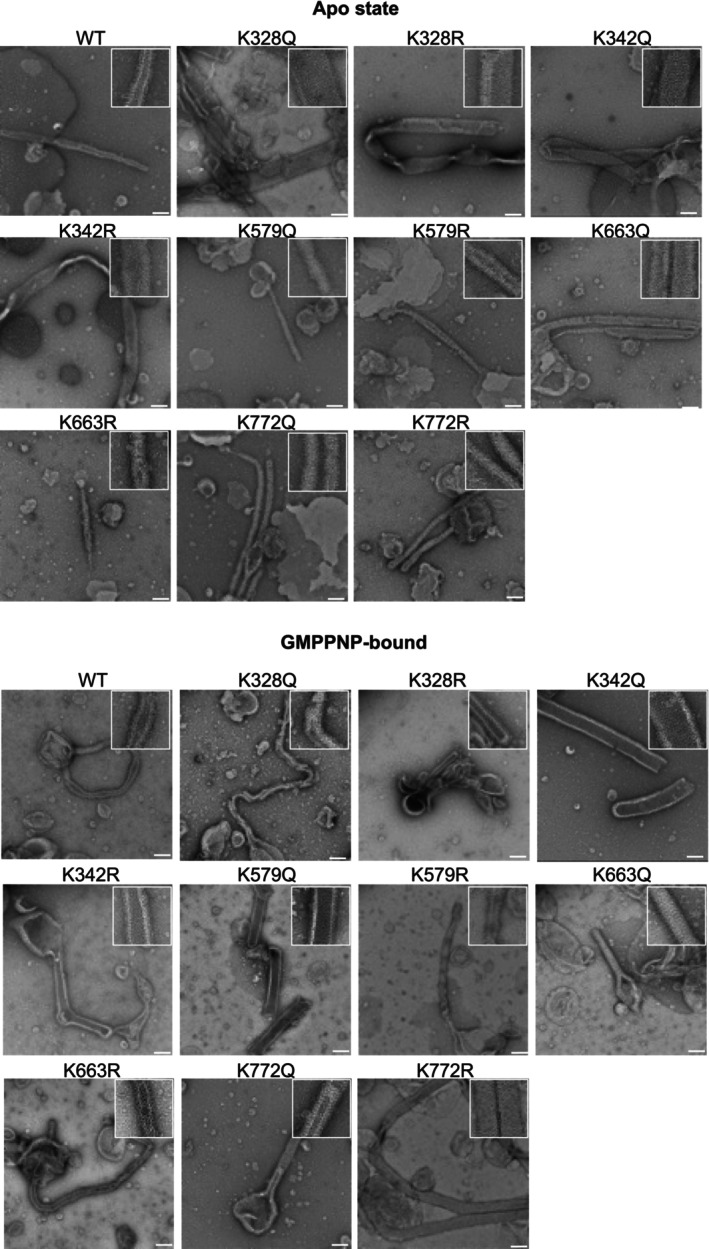
Oligomerization and liposome tubulation by s‐OPA1 WT and mutants visualized using negative‐stain electron microscopy. Electron micrographs of s‐OPA1 WT and acetylation mimetic mutants display liposome binding, s‐OPA1 oligomerization, and liposome remodeling in the presence and absence of GMPPNP. s‐OPA1 WT and mutants form distinct filament structures, reflecting differences in oligomeric states and conformational changes within the assembled polymers. Inset shows the magnified version of the micrographs. Scale bars represent 100 nm.

To assess whether lysine acetylation influences s‐OPA1‐mediated membrane remodeling, we used negative‐stain transmission electron microscopy (TEM) to examine membrane tubulation. Tubulation occurred independently of nucleotide binding, as both WT and acetylation‐mimetic mutants were capable of deforming liposomes and generating membrane tubules in the absence of GTP (Figure 4). Filaments were assessed based on their width and the organization of OPA1 oligomers. In the apo state, K328Q formed filaments approximately 125 nm in diameter with an ordered lattice, whereas K328R formed narrower filaments (~100 nm) with a disordered lattice. We did not observe any difference in the filament diameter or the lattice organization between Q and R variants at K342, K579, K663, and K772. Compared to WT producing filaments of approximately 50 nm, G domain mutants K328Q, K328R, K342Q, and K342R made wider filaments with organized lattice, with approximate diameters of 125, 100, 100, and 100 nm, respectively. The diameters and the lattice organization of filaments formed by Q and R variants of K579, K663, and K772 were similar to WT (Figure 4). In the absence of s‐OPA1, cardiolipin‐rich liposomes displayed no tubulation (Figure S4).

To further investigate the role of GTP binding in membrane tubulation, we utilized GMPPNP, a non‐hydrolysable GTP analog. Upon GMPPNP binding, both K328Q and K328R mutants formed thinner filaments (~50 nm), with K328R exhibiting a more organized lattice compared to K328Q. K342Q and K342R filaments also displayed organized lattices; however, only K342R showed a reduction in filament diameter to ~80 nm, while K342Q retained its original diameter of 100 nm. The K579R mutant showed no significant change in filament width or organization, whereas K579Q formed ~88 nm filaments with an organized lattice. Similarly, K663Q and K663R retained filament widths comparable to their apo states but demonstrated enhanced assembly, evident by lattice formation. Notably, K772R formed wider filaments (~88 nm) with organized lattices compared to the apo state, while K772Q filaments remained unchanged at 60 nm. In comparison to WT, which showed no differences between apo and GMPPNP‐bound states, K342Q/R, K579Q, and K772R mutants formed wider filaments, whereas other mutants exhibited no significant changes (Figure 4).

Our data suggests that lysine acetylation affects membrane remodeling in a position‐dependent manner. In the apo state, K328Q and K342Q/R mutants formed wider filaments, while in the pre‐hydrolysis GTP‐bound state, K342Q/R, K579Q, and K772R formed wider filaments. Although both WT and mimetic mutants were endogenously acetylated at additional lysine residues, K328Q and K772Q shared a unique modification: acetylation only at K713 (Figure 2c). This allowed for a direct comparison. In the apo state, K328Q formed significantly wider and more organized filaments than K772Q (Figure 4). Similarly, WT OPA1 was acetylated solely at K342 and formed much thinner filaments compared to K342Q in both apo and GMPPNP‐bound states. K342Q, which contains 11 additional acetylation sites (Figure 2c), formed thicker filaments than WT. This observation highlights that acetylation at different lysine residues can act in cooperation and regulate OPA1's membrane remodeling activity.

### 2.4 Characterization of GTPase activity

Previous studies have shown that the GTPase activity of OPA1 is enhanced upon binding to cardiolipin‐enriched membranes (Ban et al. 2010). Cardiolipin is crucial for maintaining mitochondrial morphology, as the interaction between OPA1 and cardiolipin is essential for IMM fusion and cristae organization (von der Malsburg et al. 2023). To elucidate the impact of lysine acetylation on OPA1 function, we assessed the GTPase activity of acetylation (Q) and deacetylation (R) mimetic mutants in the presence and absence of liposomes.

**FIGURE 5:**
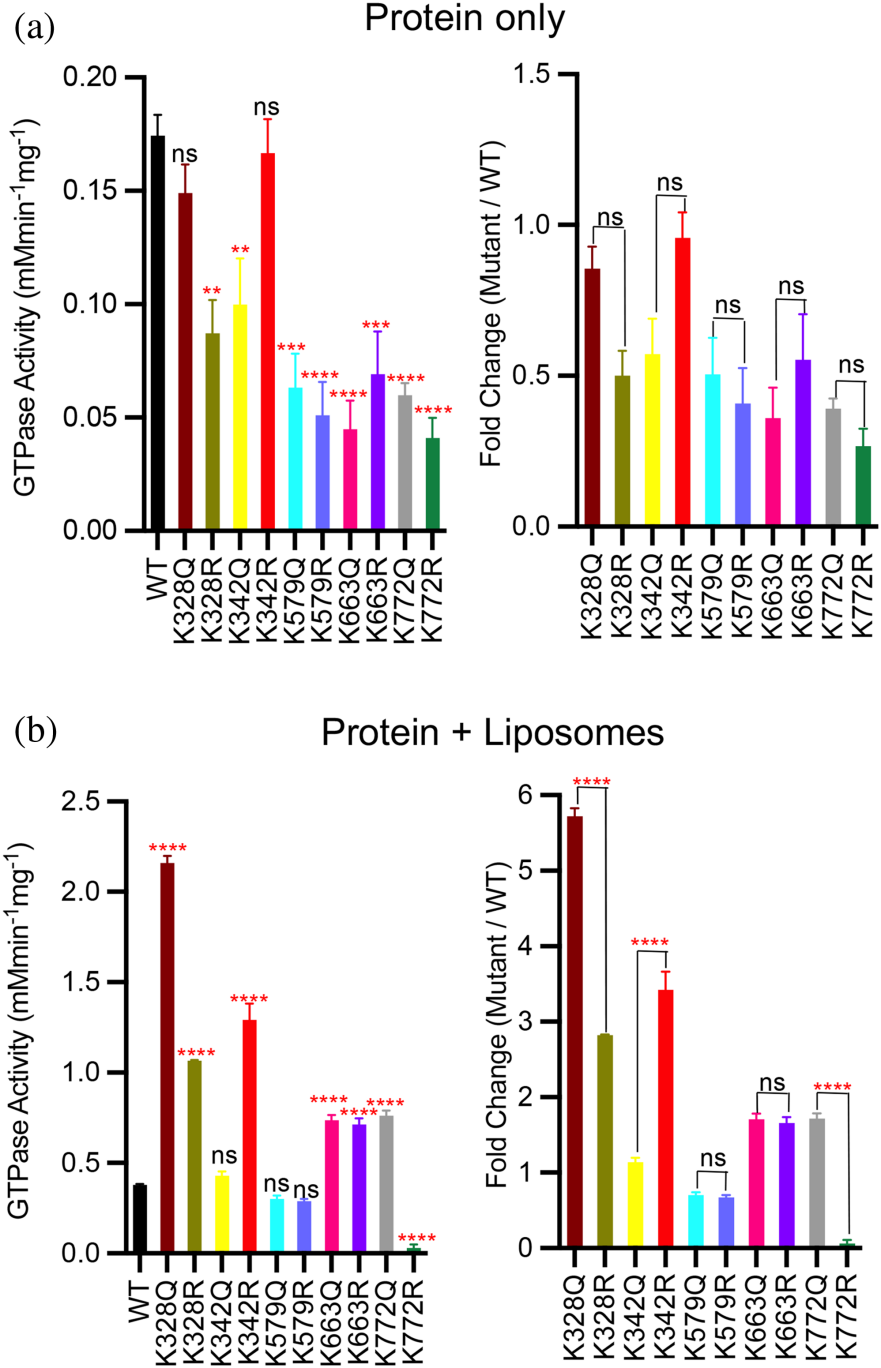
Characterization of the GTP hydrolysis activity of s‐OPA1 WT and mimetic mutants. (a) GTPase activity of s‐OPA1 and mutants at the basal level and (b) induced by liposome binding. The bar graph shows mean ± SEM of triplicates. One way ANOVA was performed and Dunnett's multiple comparisons test was conducted to compare the mean of each mutant with the wild type (left), and Tukey's multiple comparisons test was performed comparing each value with one and other (right). *p* < 0.0001 (****), *p* < 0.001 (***), *p* < 0.01 (**), ns, not significant.

In the absence of liposomes, the mimetic mutants exhibited no significant differences in GTPase activity between the Q and R forms at each lysine position tested. Compared to WT, K328Q showed no significant change in activity, while K328R displayed a ~40% reduction. Conversely, K342R retained WT‐like activity, whereas K342Q exhibited a ~30% decrease. Both Q and R mutants at K579, K663, and K772 demonstrated reduced GTPase activity relative to WT, with K772R exhibiting the lowest basal activity (Figures 5a and S5). These findings suggest that site‐specific acetylation or deacetylation induces conformational changes in the OPA1 dimer that modulate its basal GTPase activity.

Upon binding to liposomes, all mutants except K772R showed increased GTPase activity (Figures 5b and S5). When comparing Q and R variants, K328Q exhibited twice the activity of K328R, while K342R showed a threefold higher activity than K342Q. No significant differences were observed between Q and R variants at K579 and K663; however, K772Q exhibited significantly higher GTPase activity than K772R (Figure 5b, left). Compared to WT, K328Q showed the highest GTPase activity, with a sixfold increase, while K328R exhibited a 2.8‐fold increase. K342Q activity was similar to WT, whereas K342R showed a 3.5‐fold increase. Both K579Q and K579R exhibited GTPase activity comparable to WT. Although the GTPase activities of K663Q and K663R were similar to each other, both showed a ~1.7‐fold increase relative to WT. The most dramatic difference was observed at K772: K772Q exhibited a 1.7‐fold increase, while K772R showed a ~3‐fold reduction in GTPase activity upon liposome binding (Figures 5b, right and S5).

Notably, in addition to the K772R mutation, the protein contained 10 endogenous acetylated lysine residues, including K328 and K342 (Figure 2c). WT OPA1, with acetylation only at K342, exhibited one of the lowest increases in GTP hydrolysis upon liposome binding (Figure 5b). Although acetylation mimetic mutation (Q) at K328 enhanced GTPase activity (Figure 5b), co‐acetylation at K342 appeared to counteract this effect, likely by disrupting hydrogen bonds between K328 and D273 along with K342 and D277 (Figure S2b), thereby altering the conformation of the BSE, a domain known to regulate OPA1 GTPase activity (Nyenhuis et al. 2023). This suggests a regulatory mechanism in which coordinated lysine acetylation and deacetylation fine‐tune OPA1 GTPase activity, with acetylation at K342 specifically acting to reduce activity. Together, our data demonstrate that lysine acetylation regulates OPA1 function in a position‐dependent manner: acetylation mimetic mutations at K328 and K772, and the deacetylation mimetic mutation at K342, significantly enhancing GTPase activity.

### 2.5 Proposed mechanism for the role of acetylation in OPA1 mediated IMM fusion

**FIGURE 6:**
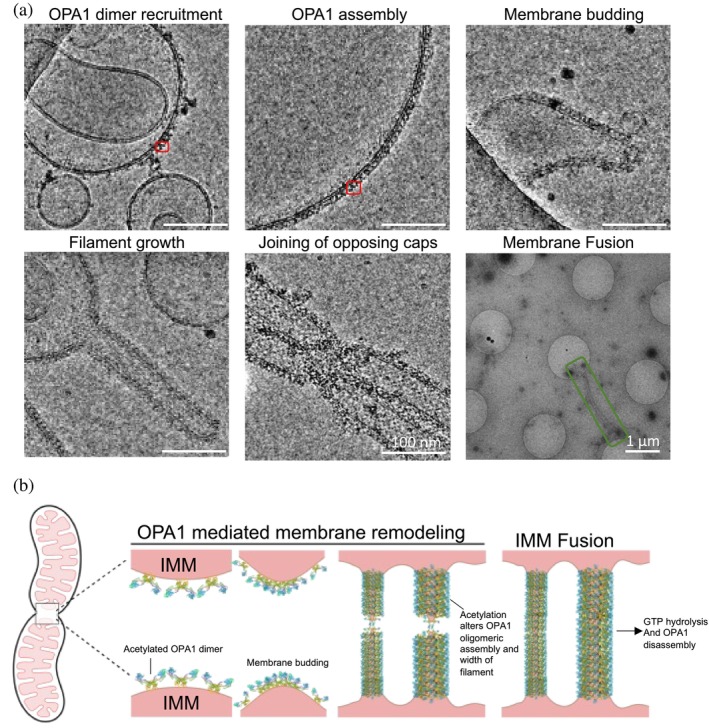
Proposed mechanism for lysine acetylation of OPA1 modulating membrane remodeling and IMM fusion. (a) Cryo‐electron micrographs depicting distinct stages of OPA1‐mediated membrane remodeling and fusion using liposomes that mimic the IMM. The acetylation mimetic mutant K328Q was used to capture these structural intermediates. The red box highlights a K328Q dimer, while the green box indicates a post‐fusion elongated filament. Scale bars represent 100 nm, except for the final micrograph (far right), which has a 1 μm scale bar. (b) Schematic model illustrating proposed sequence of events during IMM fusion. On the left, mitochondria in the early stages of fusion are shown, where outer membrane fusion brings inner membranes into proximity. Lysine acetylation induces conformational changes within the dimer which upon recruitment to the IMM facilitates further assembly resulting in membrane budding. S‐OPA1 forms helical assembly on the budding membrane enabling filament growth and lysine acetylation regulating the width of the filament. When opposing filaments meet, OPA1 molecules from each filament interact, decreasing the energy barrier at the filament caps and allowing lipid mixing. This is followed by a rearrangement of the protein subunits to establish a continuous helical assembly. Finally, GTP hydrolysis, modulated by lysine acetylation, triggers s‐OPA1 disassembly, completing the fusion process.

We used cryo‐electron microscopy (cryo‐EM) to investigate the acetylation mimetic mutant K328Q, which exhibits the highest GTPase activity, to elucidate the role of site‐specific acetylation in regulating OPA1 function and IMM fusion (Figure 6a). Previous studies have shown that the transmembrane domain of long‐form OPA1 (l‐OPA1), along with the paddle domain, interacts with the mitochondrial inner membrane, anchoring OPA1 at the fusion site and initiating the assembly of s‐OPA1 oligomers (Ban et al. 2010; Ban et al. 2017; Chan 2006; Ge et al. 2020; Nyenhuis et al. 2023). Based on cryo‐EM results, we propose a model in which acetylated s‐OPA is recruited to the IMM as a “Y” shaped dimer (Figure 6a). Subsequent assembly of these dimers induces curvature in the lipid bilayer, leading to membrane budding (Figure 6).

The acetylated s‐OPA1 then forms a helical assembly whose width varies depending on the acetylation state of OPA1 (Figure 4), facilitating filament growth (Figure 6). The width of the tube may influence the rate of filament elongation. When opposing filaments meet, OPA1 molecules from each filament interact, decreasing the energy barrier at the filament caps and allowing lipid mixing. This is followed by a rearrangement of the protein subunits to establish a continuous helical assembly. As shown elsewhere, GTP hydrolysis leads to disassembly of the protein from the lipid membrane (Nyenhuis et al. 2023), thereby recycling OPA1 into the intermembrane space. Although the structural mechanism of OPA1 disassembly from the IMM remains unclear, our data suggest that lysine acetylation regulates this process by modulating GTP hydrolysis. For instance, proteins with high GTPase activity, such as K328Q, are likely to disassemble more rapidly than those with lower activity, such as K772R.

## 3 DISCUSSION

In this study, we investigated the effects of lysine acetylation on s‐OPA1 by introducing acetylation mimetic mutations where Q and R represented acetylated and deacetylated form of lysine, respectively. We selected five sites including K328 K342, K579, K663, and K772 based on our bioinformatic analysis which suggested that acetylation at these sites may disrupt hydrogen bonding between the lysine and interacting residues, consequently affecting conformation of the protein. Our results suggest that while these modifications did not affect OPA1's ability to bind liposomes mimicking the IMM, they significantly influenced membrane remodeling and GTPase activity in a position‐dependent manner.

For instance, G‐domain acetylation mimetic mutants K328Q, K342Q, and deacetylation mimetic mutant K342R exhibited increased filament width in the apo state compared to WT. In the GTP pre‐hydrolysis state, Q and R mutants in the G‐domain for site K342 maintained this widened morphology, whereas stalk domain mutant K579Q and the paddle domain mutant K772R also displayed filament widening. This variability in filament width may influence the rate of filament elongation, thereby potentially affecting IMM fusion. Future experiments quantifying filament growth rates will be essential to determine whether the observed differences in filament width among the mutants translate to altered filament dynamics and membrane remodeling efficiency.

Notably, the width of the filament formed by s‐OPA1 did not consistently correlate with GTPase activity. While the diameters of the filaments formed by G domain mutants K328Q, K342Q, and K342R were more than WT, their GTPase activities varied: K328Q and K342R exhibited sixfold and 3.5‐fold increases, respectively, whereas K342Q showed no difference compared to WT. Conversely, the stalk domain mutants K579Q/R, K663Q/R, and the paddle domain mutant K772Q formed thin filaments like WT, yet K663Q/R and K772Q showed a 1.7‐fold increase in GTPase activity, while K579Q/R remained comparable to WT. As shown previously, GDP‐AlF_x_ binding to WT OPA1 constricts filaments, suggesting that GTP hydrolysis promotes filament narrowing and OPA1 disassembly (Nyenhuis et al. 2023). Only K579Q and K772R mutants exhibited filament widening upon GMPPNP binding, yet the GTPase activity of K579Q was similar to WT, and K772R displayed a significant decrease in GTP hydrolysis with respect to WT. Besides having other endogenously acetylated lysines, K772R was the only mutant co‐acetylated at both K328 and K342, suggesting that acetylation at these positions may disrupt intramolecular interactions between the G domain and the BSE, triggering conformational changes that modulate enzymatic activity. These findings highlight a regulatory role for lysine acetylation in modulating GTP hydrolysis, particularly within the G domain, where acetylation at K328 and deacetylation at K342 enhance OPA1 enzymatic activity.

While our study offers important insights into the role of lysine acetylation in modulating s‐OPA1 function, several limitations should be acknowledged. First, both WT and the mutant OPA1 proteins were endogenously acetylated at multiple sites, complicating direct comparisons between mutants and limiting our ability to attribute functional effects to specific acetylation events. Additionally, the use of negative stain electron microscopy (EM) presents inherent constraints. Although valuable for visualizing protein assemblies, this technique may not capture the full range of filament packing arrangements and could underrepresent the dynamic and heterogeneous nature of s‐OPA1 oligomerization. Consequently, the filament structures we observed may reflect only a subset of the possible conformational states. Future investigations employing cryo‐EM, which provides higher resolution and preserves protein assemblies in a more native context, will be crucial for resolving the structural diversity of s‐OPA1 oligomers and validating our findings with greater statistical robustness.

Given the established role of acetylation in neurodegenerative disorders and its importance in regulating protein function, including that of s‐OPA1, we examined the association of acetylation sites with known mutations linked to DOA. Among the 16 lysines known to be acetylated, mutations at four are implicated in DOA. G domain mutations such as K328R, K342E, and frameshift mutations K663N and K699N in the stalk domain are linked to DOA (Nyenhuis et al. 2023). Our data indicate that the K328R mutation shows reduced GTPase activity compared to the acetylation mimetic mutant K328Q. Our findings underscore the importance of regulating the charge on key lysine residues for the proper function of s‐OPA1. Disruptions in this acetylation process may lead to defective oligomerization, disrupted membrane remodeling, impaired GTP hydrolysis, and OPA1 disassembly, all of which are critical for maintaining functional mitochondria and may contribute to the pathogenesis of neurodegenerative diseases such as DOA.

## 4 METHODS

### 4.1 Protein expression and purification

The gene encoding the short form of human OPA1 (residues 195–960) WT and mutants were purchased from Genscript in the pGS‐21a vector. WT and mutants were transformed into *Escherichia coli* BL21 (DE3) cells. A single colony was inoculated into lysogeny broth supplemented with 100 μg mL^−1^ ampicillin and grown overnight in an orbital shaker at 37°C. A 10 mL overnight culture was used to inoculate 1 L of terrific broth medium, and cells were grown at 37°C to an optical density of 0.8–1.2. The culture was grown for another 16 h at 16°C. The cells were centrifuged at 8000 rpm for 20 min, and the pellets were stored at −80°C until further use. The cells were thawed and resuspended in buffer A (20 mM HEPES‐NaOH, pH 7.5, 500 mM NaCl, 5 mM CHAPS, 5 mM 2‐mercaptoethanol, 10% (v/v) glycerol), 0.01% DNase I, 1× EDTA‐free complete protease inhibitor cocktail (Roche) and 1 mg mL^−1^ lysozyme. Cells were lysed using a probe sonicator and centrifuged at 18,000 rpm at 4°C for 45 min to remove cell debris. Using the 0.45 μm membrane, the supernatant was filtered and loaded onto glutathione resin pre‐equilibrated with buffer A and rotated for 2 h at 4°C. The beads were washed with 10 column volumes of buffer A followed by elution with buffer B (20 mM HEPES‐NaOH, pH 7.5, 300 mM NaCl, 5 mM MgCl_2_, 5 mM CHAPS, 5 mM 2‐mercaptoethanol, 10% (v/v) glycerol, and 10 mM L‐Glutathione (reduced)). The eluate was buffer exchanged with buffer A and incubated with 3C protease overnight at 4°C to cleave the GST tag. The sample was applied again to glutathione resin pre‐equilibrated with buffer A to remove un‐cleaved protein, free purification tag, and the protease. The cleaved protein was purified further using size exclusion chromatography using Superdex‐200 16/60 (Cytiva) equilibrated with buffer C (20 mM HEPES‐NaOH, pH 7.5, 500 mM NaCl, 5 mM MgCl_2_, 5 mM CHAPS, 5 mM 2‐mercaptoethanol, and 10% (v/v) glycerol). Peak fractions were analyzed using SDS‐PAGE analysis, pooled, and concentrated using a 30 kDa MWCO filter (amicon). Proteins were flash frozen to be used as single‐use aliquots and kept at −80°C.

### 4.2 Mass photometry

The movies were recorded on the Refeyn mass photometer. Gaskets were cleaned using isopropanol, rinsed with ddH_2_O, and dried using a stream of nitrogen. Protein sample buffer (20 mM HEPES‐NaOH, pH 7.5, 130 mM NaCl, 10 mM KCl, 2 mM MgCl_2_, 2 mM DTT, 2% (v/v) glycerol) was filtered through a 0.22 μm membrane, followed by protein sample dilutions to 150 nM. A dried gasket was assembled onto a clean coverslip and placed onto the mass photometer. Instrument calibration was determined using contrast to mass calibration with bovine serum albumin and thyroglobulin. Data was acquired using AcquireMP 2.2 software (Refeyn) and analyzed with DiscoverMP 2.2 software (Refeyn). Ten μL of protein samples were applied to 10 μL of sample buffer on the coverslip, yielding a final concentration of 75 nM. Mass photometry videos were acquired for 60 s, followed by the quantification of individual particle binding events and mass conversion of each video using instrument calibration. The output is a mass distribution of individual binding events demonstrating a distribution of oligomeric species in the sample solution. The difference between measured data and the linear fit of calibration is assessed by the software to calculate the accuracy of the measured mass. Mass was measured three times for each protein.

### 4.3 Preparation of liposomes

The following lipids were purchased from Avanti Polar Lipids: 1‐palmitoyl‐2‐oleoyl‐sn‐glycero‐3‐phosphocholine (POPC), 1‐palmitoyl‐2‐oleoyl‐sn‐glycero‐3‐phosphoethanolamine (POPE), L‐α‐lysophosphatidylinositol (PI), and 1′,3′‐bis[1,2‐dioleoyl‐sn‐glycero‐3‐phospho]‐glycerol (cardiolipin). Stock lipids were resuspended in a mixture of chloroform, methanol, and water (20:9:1 (v/v/v)). The lipid composition of liposomes (POPC:POPE:PI:CL; 45:22:8:25) was prepared to represent the lipid composition of the inner membrane of mitochondria (Chan 2006). The required ratio of lipid stock solutions was mixed in glass vials and dried under a steady stream of nitrogen while rotating the vial to obtain homogeneous thin lipid films. Dehydrated lipids were placed inside a vacuum desiccator for 4 h to remove the residual chloroform. The resulting lipid monolayers were rehydrated in buffer according to the experiments: GTPase and co‐sedimentation assay (20 mM HEPES‐NaOH, pH 7.5, 150 mM NaCl, 2 mM MgCl_2_, 2.5 mM TCEP) and liposome tubulation assay (20 mM HEPES‐NaOH, pH 7.5, 130 mM NaCl, 10 mM KCl, 2 mM MgCl_2_, 2 mM DTT, 2% (v/v) glycerol). The hydrated lipids were vortexed, subjected to five freeze–thaw cycles, and extruded through a 0.8 μm polycarbonate membrane to yield unilamellar liposomes. Lipid vesicles were kept in glass vials at 4°C in the dark and used within a week.

### 4.4 Liposome tubulation assay

WT and mutant s‐OPA1 proteins were buffer exchanged to assay buffer (20 mM HEPES‐NaOH, pH 7.5, 130 mM NaCl, 10 mM KCl, 2 mM MgCl_2_, 2 mM DTT, 2% (v/v) glycerol). Two μM protein was incubated with 0.5 mg mL^−1^ liposomes in the absence or presence of 1 mM GMPPNP at room temperature for 3 h. The samples were immediately processed for negative staining electron microscopy sample preparation.

### 4.5 Liposome co‐sedimentation assays

WT and mutant s‐OPA1 proteins were buffer exchanged to liposome buffer (20 mM HEPES‐NaOH, pH 7.5, 150 mM NaCl, 2 mM MgCl_2_, 2.5 mM TCEP) using 50 MWCO filters (amicon) at 4°C. One micromolar s‐OPA1 WT and mutants were incubated with 0.5 mg mL^−1^ liposomes for 30 min at room temperature. Protein only did not contain any lipid vesicles. The samples were centrifuged at 55,000 rpm at 20°C for 30 min using a TLA‐100 rotor (Beckman Coulter) and the pellet and supernatant were analyzed using SDS‐PAGE. The experiment was performed in triplicates. Gel bands were quantified using ImageJ (Schindelin et al. 2012).

### 4.6 Negative staining electron microscopy

4 μL of sample solution prepared for liposome tubulation was applied onto a glow‐discharged 200 mesh copper grid containing a thin layer of carbon film. The grids were blotted with filter paper, rinsed with water, and stained with 1% (w/v) uranyl acetate for 60 s. Excess stain was removed using a filter paper, and the samples were visualized at room temperature using a Talos 120C microscope (ThermoFisher), equipped with a 4 k × 4 k ceta CMOS camera and operated at a voltage of 120 kV. Micrographs were acquired at a magnification of ×57,000 with a calibrated pixel size of 2.44 Å. A minimum of 10 images were acquired for each sample.

### 4.7 Cryo‐electron microscopy sample preparation, data acquisition, and processing

4 μL of sample of K328Q prepared for liposome tubulation was applied onto 30 s glow discharged R1.2/1.3200 Cu mesh grids (Quantifoil). The sample was incubated for 30 s. The grids were blotted for 4 s at blot force 0 and plunge frozen in liquid ethane using Vitrobot Mark IV (FEI), operated at 4°C and 100% humidity. Movies were recorded on Titan Krios G3 microscope (ThermoFisher Scientific) operated at 300 kV, equipped with Falcon 4i camera. A total of 7448 movies were recorded at a magnification of 96,000×, with a calibrated pixel size of 0.808 Å, defocus range of 0.6–1.6 μm, 40 frames, and 38.7 e^−^/Å^2^ electron exposure per movie. Gain correction and motion correction were done using cryoSPARC v4.5.3 (Punjani et al. 2017).

### 4.8 GTPase assays

WT and mutants were buffers exchanged to GTPase assay buffer (20 mM HEPES‐NaOH, pH 7.5, 150 mM NaCl, 2 mM MgCl_2_, 2.5 mM TCEP) using 50 MWCO filters (amicon) at 4°C. The reactions were performed with 0.1 mg mL^−1^ protein and for liposome‐induced GTPase reactions, 0.1 mg mL^−1^ liposomes were added on a 96‐well plate. Proteins were incubated at 37°C for 30 min before initiating the reaction by the addition of 1 mM GTP. The reactions were further incubated at 37°C for another 60 min and quenched using 12.5 μL 0.5M EDTA, pH 8. Fifty microliter of Malachite green reagent per well was added followed by the addition of 5 μL 34% (w/v) sodium citrate. The reactions were covered using aluminum foil and after 30 min of incubation at room temperature, absorbance at 650 nm was recorded using a plate reader (Clariostar). The free phosphate concentration was determined by the standard curve plotted using the phosphate standards. Blank containing GTP in the GTPase buffer was subtracted from each reaction to account for the self‐hydrolysis of GTP. GTPase activity of protein was determined using the equation ([Pi] produced (mM)/reaction time (min) × amount of protein (mg)). Fold change was determined by dividing the GTPase activity of the mutant by WT s‐OPA1. The data was plotted as a bar graph showing mean ± SEM of triplicates. For statistical analysis, one‐way ANOVA followed by Dunnett's multiple comparisons test for GTPase activity and Tukey's multiple comparisons test for fold change was performed in GraphPad Prism. The bar graphs were also plotted in GraphPad Prism.

### 4.9 In silico analysis of s‐OPA1

The predicted Gibbs free energy (ΔΔ*G*) for point mutations was determined using DynaMut2 (Rodrigues et al. 2021) available at http://biosig.unimelb.edu.au/dynamut2. A mutation list with a point mutation in both chains of the dimer was given as input along with PDB id for apostate and GDP‐AlF_x_ bound state: 8EF7 and 8EEW (Nyenhuis et al. 2023), respectively. The output ΔΔ*G* was plotted using GraphPad Prism. For interatomic bond analysis, published cryoEM structures for apostate (PDB: 8EF7) and GDP‐ALF_x_ bound (PDB: 8EEW) were analyzed using ChimeraX (Meng et al. 2023). The domains were color‐coded according to the domain model in Figure 1a, and chain B was colored in lighter hues. Hydrogen bonds, if present, were shown with a dotted line in red color. The representative residues were colored in heteroatom and shown as sticks.

Sequence alignment of OPA1 was performed using clustal omega v1.2.4 (Sievers and Higgins 2018). OPA1 protein sequences of *Homo sapiens* (NP_056375.2), *Mus musculus* (NP_598513.1), and *Rattus norvegicus* (NP_598269.3) were aligned and ESpript 3.0 (Robert and Gouet 2014) was used to generate the image. Lysine residues that get acetylated in OPA1 in different organisms were determined using the PhosphoSitePlus v6.7.5 server (Hornbeck et al. 2015).

### 4.10 Mass spectrometry analysis

Two microgram protein was loaded onto polyacrylamide gels followed by SDS‐PAGE. The gel was stained using Coomassie dye and de‐stained using 50% methanol, 40% ddH_2_O, and 10% acetic acid. Gel spots were de‐stained for 2 h at 37°C using de‐staining solution (50% Acetonitrile (ACN) in 50 mM Triethylammonium bicarbonate (TEAB)). After removing the de‐staining solution, the gel spots were incubated with 100% ACN for 10 min, air dried, and incubated with 50 mM TCEP solution (ThermoFisher) for 1 h at 60°C. After removing TCEP, 100 mM iodoacetamide was added and samples were incubated for 30 min in the dark at room temperature. Iodoacetamide was removed and gel spots were washed using de‐staining solution followed by gel dehydration with 100% ACN. The gel pieces were air dried and digested with trypsin (1:50 enzyme: protein) overnight at 37°C. The digested peptides were analyzed on a Thermo Q Exactive™ Plus mass spectrometer coupled with a Dionex Ultimate 3000 HPLC. Raw files were analyzed using MaxQuant version 2.2.0.0 (Cox and Mann 2008) and searched against the Uniprot human database containing reviewed, canonical variants in FASTA format (March, 2022) and Adromeda search engine database (Cox et al. 2011) containing common contaminants. Default search parameters for label‐free quantification (LFQ) were used with a few modifications. Briefly, cysteine carbamidomethylation was used as a fixed modification and N‐terminal acetylation, methionine oxidation, and lysine acetylation were used as variable modifications. False discovery rate of 1% was applied, and match between runs was enabled with a match time window of 0.7 min.

## AUTHOR CONTRIBUTIONS

**Javaid Jabbar:** Conceptualization; investigation; funding acquisition; writing – original draft; methodology; validation; formal analysis. **Bakht Afroze:** Investigation; methodology; writing – original draft; resources. **Naomi X. Y. Ling:** Investigation; methodology. **Jonathan S. Oakhill:** Supervision; funding acquisition; writing – review and editing. **Isabelle Rouiller:** Conceptualization; funding acquisition; writing – review and editing; project administration; supervision.

## CONFLICT OF INTEREST STATEMENT

The authors declare no conflicts of interest.

## Supporting information


**Figure S1.** Supporting Information.

## Data Availability

The data that support the findings of this study are available from the corresponding author upon reasonable request.

## References

[pro70179-bib-0001] Alexander C , Votruba M , Pesch UEA , Thiselton DL , Mayer S , Moore A , et al. OPA1, encoding a dynamin‐related GTPase, is mutated in autosomal dominant optic atrophy linked to chromosome 3q28. Nat Genet. 2000;26:211–215. 11017080 10.1038/79944

[pro70179-bib-0002] Ali I , Conrad RJ , Verdin E , Ott M . Lysine acetylation goes global: from epigenetics to metabolism and therapeutics. Chem Rev. 2018;118:1216–1252. 29405707 10.1021/acs.chemrev.7b00181 PMC6609103

[pro70179-bib-0003] Anand R , Wai T , Baker MJ , Kladt N , Schauss AC , Rugarli E , et al. The i‐AAA protease YME1L and OMA1 cleave OPA1 to balance mitochondrial fusion and fission. J Cell Biol. 2014;204(6):919–929. 10.1083/jcb.201308006 24616225 PMC3998800

[pro70179-bib-0004] Anderson KA , Hirschey MD . Mitochondrial protein acetylation regulates metabolism. Essays Biochem. 2012;52:23–35. 22708561 10.1042/bse0520023 PMC3872051

[pro70179-bib-0005] Baeza J , Smallegan MJ , Denu JM . Site‐specific reactivity of nonenzymatic lysine acetylation. ACS Chem Biol. 2015;10:122–128. 25555129 10.1021/cb500848p PMC4301072

[pro70179-bib-0006] Baeza J , Smallegan MJ , Denu JM . Mechanisms and dynamics of protein acetylation in mitochondria. Trends Biochem Sci. 2016;41:231–243. 26822488 10.1016/j.tibs.2015.12.006 PMC4783225

[pro70179-bib-0007] Ban T , Heymann JAW , Song Z , Hinshaw JE , Chan DC . OPA1 disease alleles causing dominant optic atrophy have defects in cardiolipin‐stimulated GTP hydrolysis and membrane tubulation. Hum Mol Genet. 2010;19:2113–2122. 20185555 10.1093/hmg/ddq088 PMC2865371

[pro70179-bib-0008] Ban T , Ishihara T , Kohno H , Saita S , Ichimura A , Maenaka K , et al. Molecular basis of selective mitochondrial fusion by heterotypic action between OPA1 and cardiolipin. Nat Cell Biol. 2017;19:856–863. 28628083 10.1038/ncb3560

[pro70179-bib-0009] Belenguer P , Pellegrini L . The dynamin GTPase OPA1: more than mitochondria? Biochim Biophys Acta. 2013;1833:176–183. 22902477 10.1016/j.bbamcr.2012.08.004

[pro70179-bib-0010] Bonneau D , Colin E , Oca F , Ferré M , Chevrollier A , Guéguen N , et al. Early‐onset Behr syndrome due to compound heterozygous mutations in OPA1. Brain. 2014;137:e301. 25012220 10.1093/brain/awu184

[pro70179-bib-0011] Carelli V , Musumeci O , Caporali L , Zanna C , la Morgia C , del Dotto V , et al. Syndromic Parkinsonism and dementia associated with OPA1 missense mutations. Ann Neurol. 2015;78:21–38. 25820230 10.1002/ana.24410 PMC5008165

[pro70179-bib-0012] Chan DC . Mitochondrial fusion and fission in mammals. Annu Rev Cell dev Biol. 2006;22:79–99. 16704336 10.1146/annurev.cellbio.22.010305.104638

[pro70179-bib-0013] Chen L , Liu T , Tran A , Lu X , Tomilov AA , Davies V , et al. OPA1 mutation and late‐onset cardiomyopathy: mitochondrial dysfunction and mtDNA instability. J Am Heart Assoc. 2012;1:e003012. 23316298 10.1161/JAHA.112.003012 PMC3541627

[pro70179-bib-0014] Chen W , Zhao H , Li Y . Mitochondrial dynamics in health and disease: mechanisms and potential targets. Signal Transduct Target Ther. 2023;8:333. 37669960 10.1038/s41392-023-01547-9 PMC10480456

[pro70179-bib-0015] Choudhary C , Weinert BT , Nishida Y , Verdin E , Mann M . The growing landscape of lysine acetylation links metabolism and cell signalling. Nat Rev Mol Cell Biol. 2014;15:536–550. 25053359 10.1038/nrm3841

[pro70179-bib-0016] Cohen TJ , Hwang AW , Restrepo CR , Yuan CX , Trojanowski JQ , Lee VMY . An acetylation switch controls TDP‐43 function and aggregation propensity. Nat Commun. 2015;6:5845. 25556531 10.1038/ncomms6845 PMC4407365

[pro70179-bib-0017] Cox J , Mann M . MaxQuant enables high peptide identification rates, individualized ppb‐range mass accuracies and proteome‐wide protein quantification. Nat Biotechnol. 2008;26:1367–1372. 19029910 10.1038/nbt.1511

[pro70179-bib-0018] Cox J , Neuhauser N , Michalski A , Scheltema RA , Olsen JV , Mann M . Andromeda: a peptide search engine integrated into the MaxQuant environment. J Proteome Res. 2011;10(4):1794–1805. 21254760 10.1021/pr101065j

[pro70179-bib-0019] Del Dotto V , Fogazza M , Carelli V , Rugolo M , Zanna C . Eight human OPA1 isoforms, long and short: What are they for? Biochim Biophys Acta. 2018;1859:263–269. 10.1016/j.bbabio.2018.01.005 29382469

[pro70179-bib-0020] Del Dotto V , Mishra P , Vidoni S , Fogazza M , Maresca A , Caporali L , et al. OPA1 isoforms in the hierarchical organization of mitochondrial functions. Cell Rep. 2017;19:2557–2571. 28636943 10.1016/j.celrep.2017.05.073

[pro70179-bib-0021] Delettre C , Lenaers G , Griffoin JM , Gigarel N , Lorenzo C , Belenguer P , et al. Nuclear gene OPA1, encoding a mitochondrial dynamin‐related protein, is mutated in dominant optic atrophy. Nat Genet. 2000;26(4):207–210. 11017079 10.1038/79936

[pro70179-bib-0022] Drazic A , Myklebust LM , Ree R , Arnesen T . The world of protein acetylation. Biochim Biophys Acta. 2016;1864:1372–1401. 27296530 10.1016/j.bbapap.2016.06.007

[pro70179-bib-0023] Ge Y , Shi X , Boopathy S , McDonald J , Smith AW , Chao LH . Two forms of OPA1 cooperate to complete fusion of the mitochondrial inner membrane. eLife. 2020;9:e50973. 31922487 10.7554/eLife.50973 PMC7299343

[pro70179-bib-0024] Hornbeck PV , Zhang B , Murray B , Kornhauser JM , Latham V , Skrzypek E . PhosphoSitePlus, 2014: mutations, PTMs and recalibrations. Nucleic Acids Res. 2015;43:D512–D520. 25514926 10.1093/nar/gku1267 PMC4383998

[pro70179-bib-0025] MacVicar T , Langer T . OPA1 processing in cell death and disease—the long and short of it. J Cell Sci. 2016;129:2297–2306. 27189080 10.1242/jcs.159186

[pro70179-bib-0026] Mattson MP . Methylation and acetylation in nervous system development and neurodegenerative disorders. Ageing Res Rev. 2003;2:329–342. 12726778 10.1016/s1568-1637(03)00013-8

[pro70179-bib-0027] Meng EC , Goddard TD , Pettersen EF , Couch GS , Pearson ZJ , Morris JH , et al. UCSF ChimeraX: tools for structure building and analysis. Protein Sci. 2023;32:e4792. 37774136 10.1002/pro.4792 PMC10588335

[pro70179-bib-0028] Min SW , Cho SH , Zhou Y , Schroeder S , Haroutunian V , Seeley WW , et al. Acetylation of tau inhibits its degradation and contributes to tauopathy. Neuron. 2010;67:953–966. 20869593 10.1016/j.neuron.2010.08.044 PMC3035103

[pro70179-bib-0029] Novak K , Flöckner L , Erian AM , Freitag P , Herwig C , Pflügl S . Characterizing the effect of expression of an acetyl‐CoA synthetase insensitive to acetylation on co‐utilization of glucose and acetate in batch and continuous cultures of *E. coli* W. Microb Cell Fact. 2018;17:109. 29986728 10.1186/s12934-018-0955-2 PMC6036698

[pro70179-bib-0030] Nyenhuis SB , Wu X , Strub MP , Yim YI , Stanton AE , Baena V , et al. OPA1 helical structures give perspective to mitochondrial dysfunction. Nature. 2023;620:1109–1116. 37612506 10.1038/s41586-023-06462-1 PMC12410014

[pro70179-bib-0031] Paik WK , Pearson D , Lee HW , Kim S . Nonenzymatic acetylation of histones with acetyl‐CoA. Biochim Biophys Acta. 1970;213:513–522. 5534125 10.1016/0005-2787(70)90058-4

[pro70179-bib-0032] Punjani A , Rubinstein JL , Fleet DJ , Brubaker MA . cryoSPARC: algorithms for rapid unsupervised cryo‐EM structure determination. Nat Methods. 2017;14:290–296. 28165473 10.1038/nmeth.4169

[pro70179-bib-0033] Robert X , Gouet P . Deciphering key features in protein structures with the new ENDscript server. Nucleic Acids Res. 2014;42(W1):W320–W324. 24753421 10.1093/nar/gku316 PMC4086106

[pro70179-bib-0034] Rodrigues CHM , Pires DEV , Ascher DB . DynaMut2: assessing changes in stability and flexibility upon single and multiple point missense mutations. Protein Sci. 2021;30:60–69. 32881105 10.1002/pro.3942 PMC7737773

[pro70179-bib-0035] Samant SA , Zhang HJ , Hong Z , Pillai VB , Sundaresan NR , Wolfgeher D , et al. SIRT3 deacetylates and activates OPA1 to regulate mitochondrial dynamics during stress. Mol Cell Biol. 2014;34:807–819. 24344202 10.1128/MCB.01483-13 PMC4023816

[pro70179-bib-0036] Schindelin J , Arganda‐Carreras I , Frise E , Kaynig V , Longair M , Pietzsch T , et al. Fiji: an open‐source platform for biological‐image analysis. Nat Methods. 2012;9:676–682. 22743772 10.1038/nmeth.2019 PMC3855844

[pro70179-bib-0037] Sievers F , Higgins DG . Clustal omega for making accurate alignments of many protein sequences. Protein Sci. 2018;27:135–145. 28884485 10.1002/pro.3290 PMC5734385

[pro70179-bib-0038] Tilokani L , Nagashima S , Paupe V , Prudent J . Mitochondrial dynamics: overview of molecular mechanisms. Essays Biochem. 2018;62:341–360. 30030364 10.1042/EBC20170104 PMC6056715

[pro70179-bib-0039] von der Malsburg A , Sapp GM , Zuccaro KE , von Appen A , Moss FR III , Kalia R , et al. Structural mechanism of mitochondrial membrane remodelling by human OPA1. Nature. 2023;620:1101–1108. 37612504 10.1038/s41586-023-06441-6 PMC10875962

[pro70179-bib-0040] Wagner GR , Hirschey MD . Nonenzymatic protein acylation as a carbon stress regulated by sirtuin deacylases. Mol Cell. 2014;54:5–16. 24725594 10.1016/j.molcel.2014.03.027 PMC4040445

[pro70179-bib-0041] Wang R , Mishra P , Garbis SD , Moradian A , Sweredoski MJ , Chan DC . Identification of new OPA1 cleavage site reveals that short isoforms regulate mitochondrial fusion. Mol Biol Cell. 2021;32:157–168. 33237841 10.1091/mbc.E20-09-0605 PMC8120690

[pro70179-bib-0042] Weinert BT , Iesmantavicius V , Moustafa T , Schölz C , Wagner SA , Magnes C , et al. Acetylation dynamics and stoichiometry in *Saccharomyces cerevisiae* . Mol Syst Biol. 2014;10(1):716. 10.1002/msb.134766 24489116 PMC4023402

[pro70179-bib-0043] Yu‐Wai‐Man P , Votruba M , Burté F , la Morgia C , Barboni P , Carelli V . A neurodegenerative perspective on mitochondrial optic neuropathies. Acta Neuropathol. 2016;132:789–806. 27696015 10.1007/s00401-016-1625-2 PMC5106504

[pro70179-bib-0044] Zerem A , Yosovich K , Rappaport YC , Libzon S , Blumkin L , Ben‐Sira L , et al. Metabolic stroke in a patient with bi‐allelic OPA1 mutations. Metab Brain Dis. 2019;34:1043–1048. 30972688 10.1007/s11011-019-00415-2

[pro70179-bib-0045] Zhang A , Pan Y , Wang H , Ding R , Zou T , Guo D , et al. Excessive processing and acetylation of OPA1 aggravate age‐related hearing loss via the dysregulation of mitochondrial dynamics. Aging Cell. 2024;23:e14091. 38267829 10.1111/acel.14091 PMC11019136

[pro70179-bib-0046] Zhang D , Zhang Y , Ma J , Zhu C , Niu T , Chen W , et al. Cryo‐EM structures of S‐OPA1 reveal its interactions with membrane and changes upon nucleotide binding. Elife. 2020;9:e50294. 32228866 10.7554/eLife.50294 PMC7156267

