# Supplementary material for: Lysine acetylation modulates s‐OPA1 GTPase activity and oligomerization in mitochondrial membrane remodeling
Source: Protein Sci. 2025 May 29;34(6):e70179. doi: 10.1002/pro.70179 (PMC12120360; doi:10.1002/pro.70179)
Supplement: Supplementary file 1 — Figure S1. Supporting Information. [file PRO-34-e70179-s001.docx]

Supplementary Figure 1


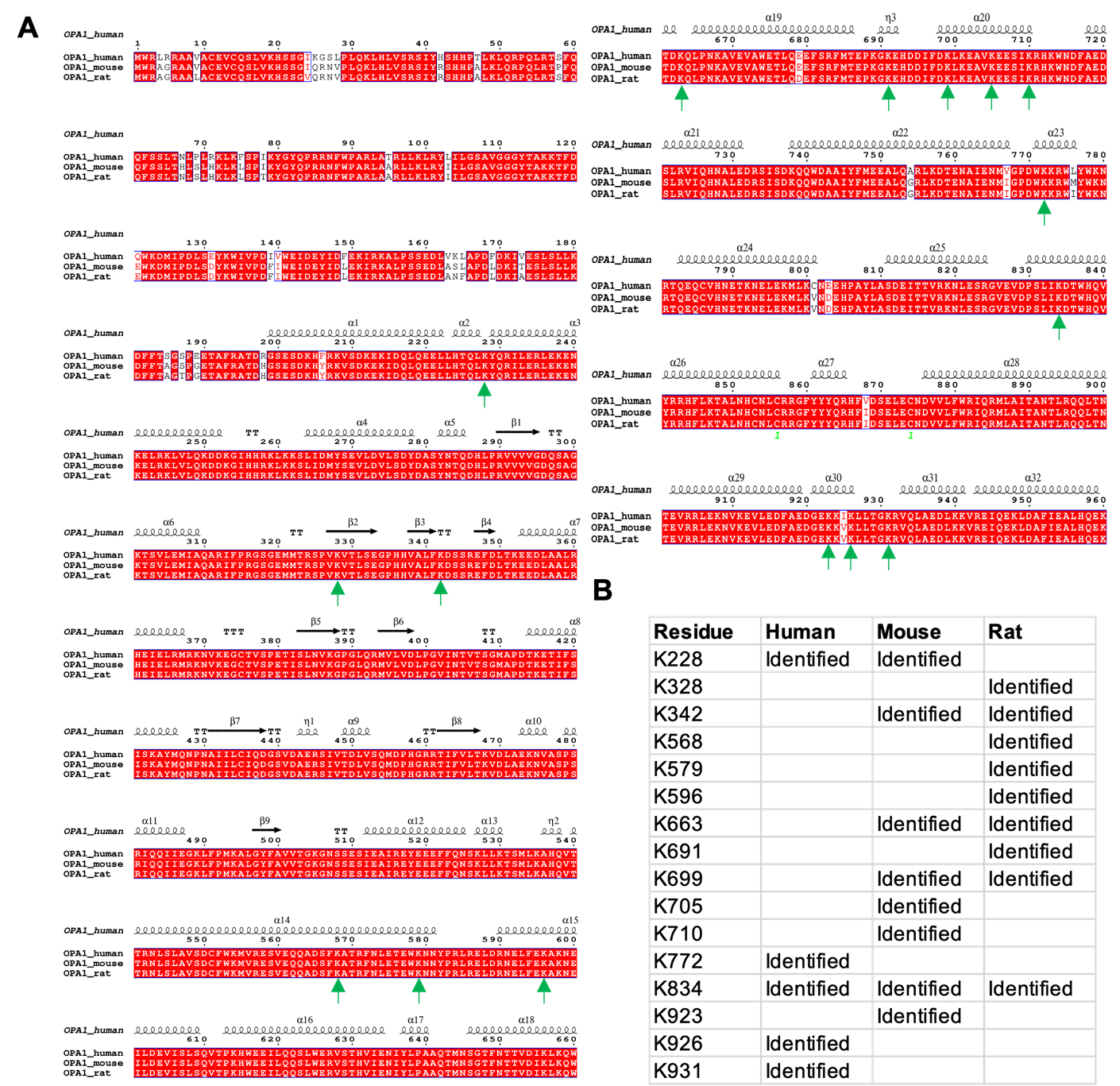


Supplementary Figure 2


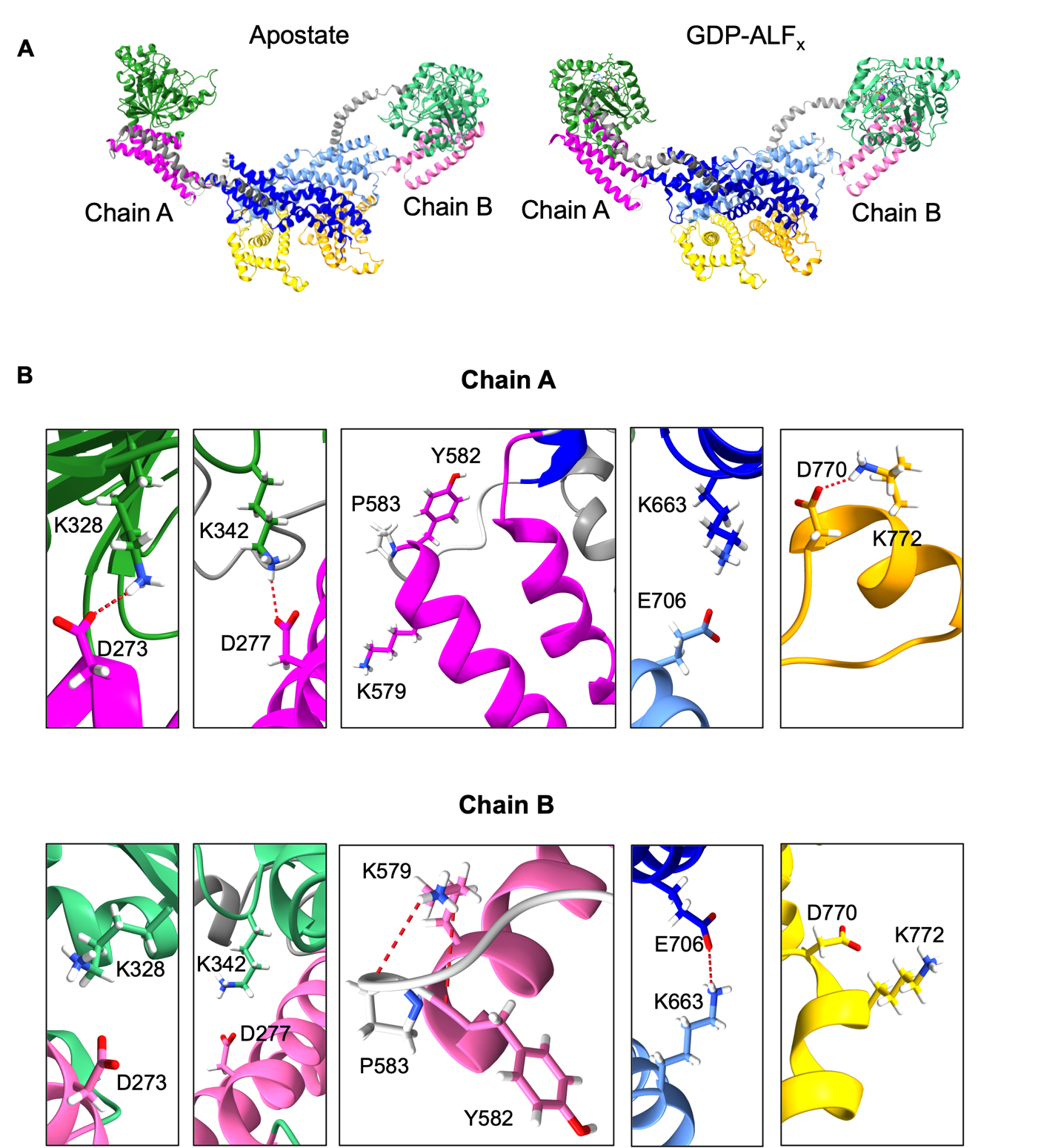


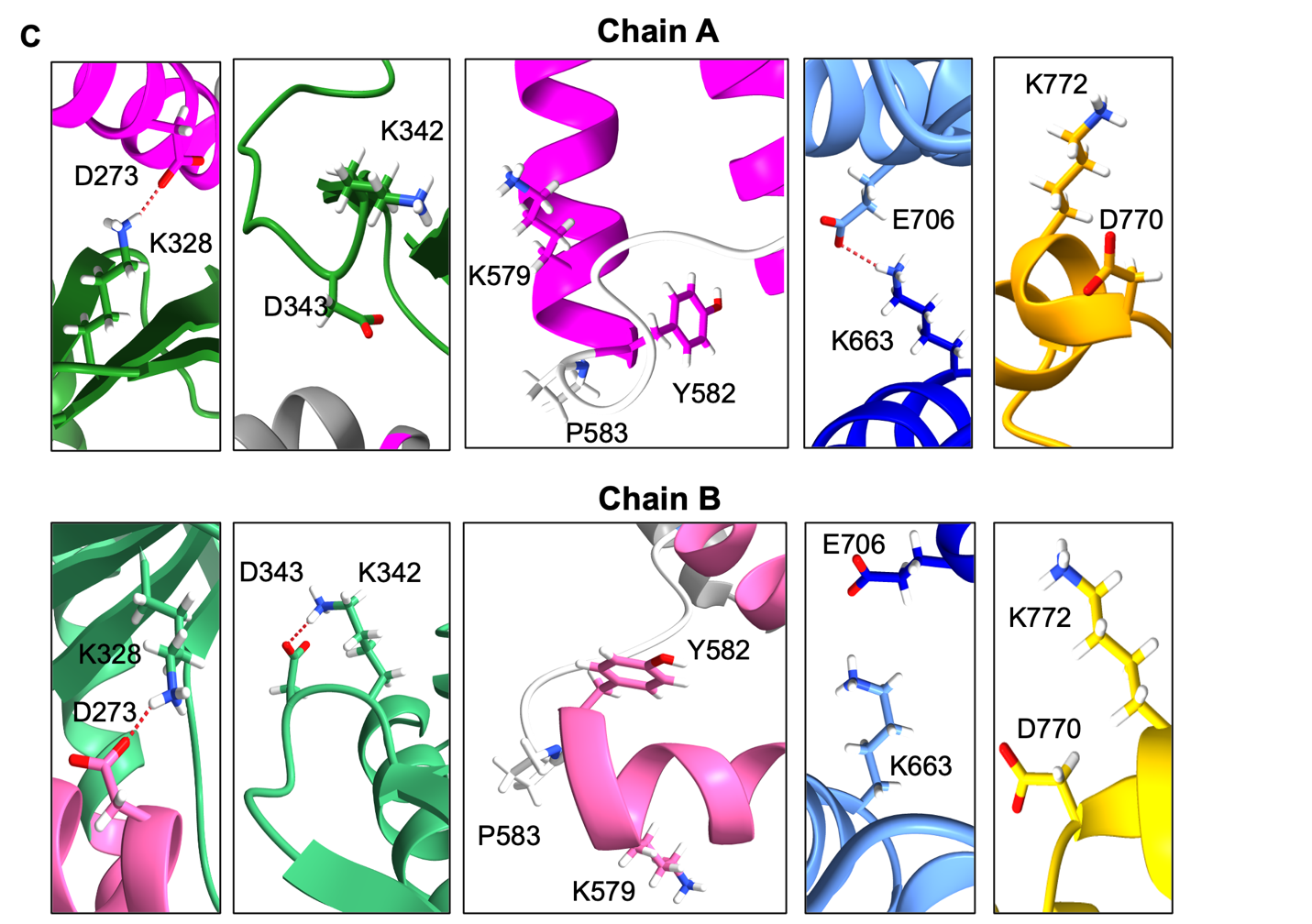


Supplementary Figure 3


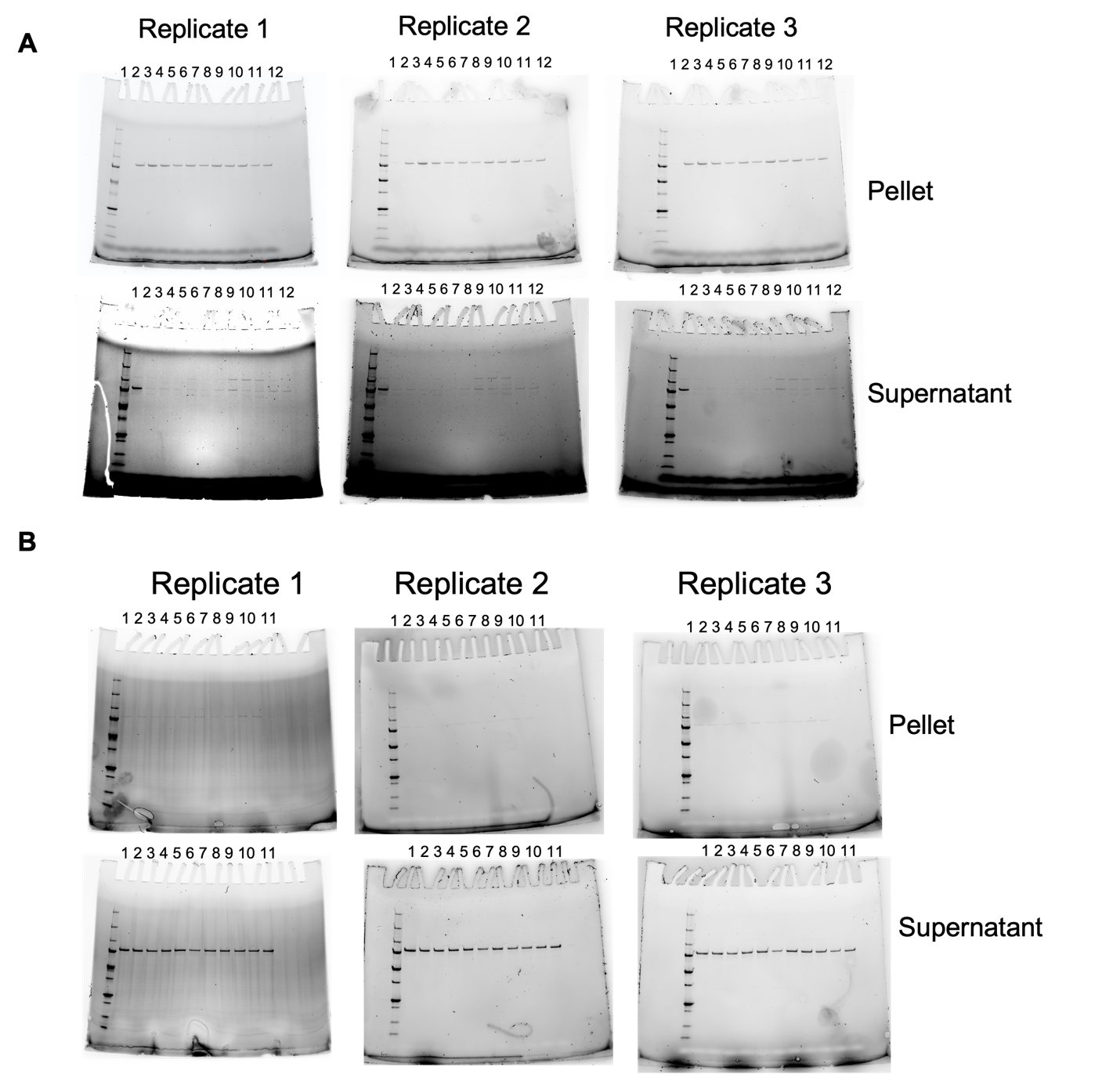


Supplementary Figure 4


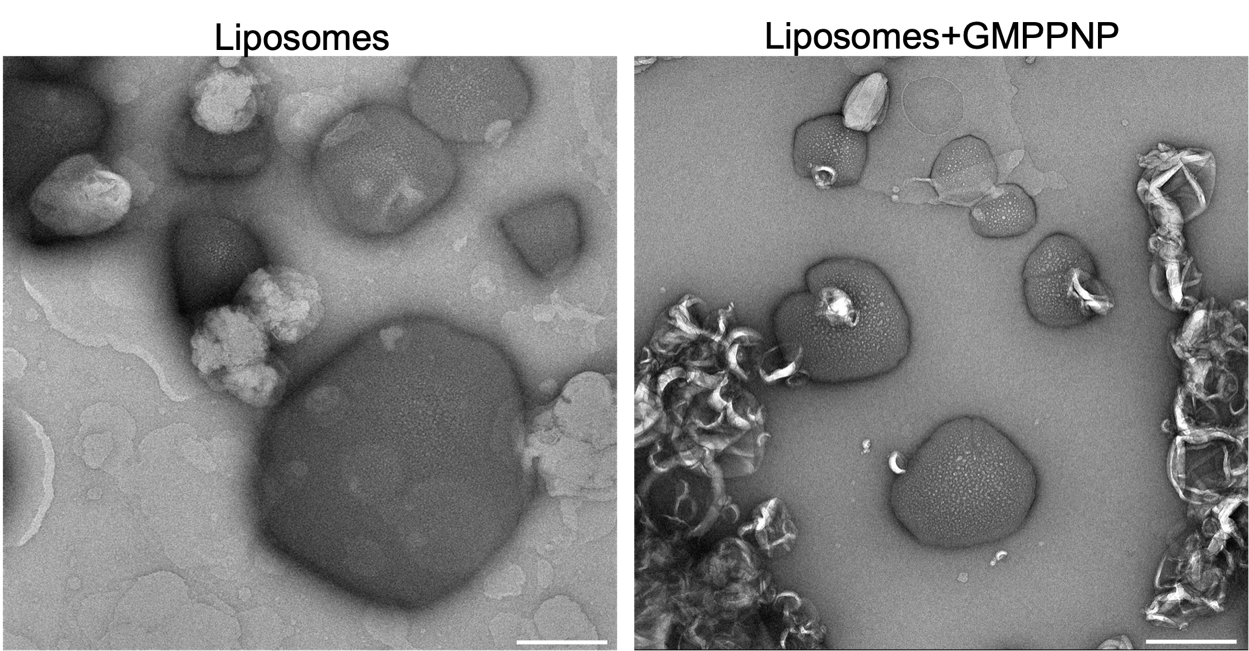


Supplementary Figure 5

**SUPPLEMENTARY FIGURE LEGENDS**

**Supplementary Figure 1: Conservation of lysine residues in OPA1. A)** Sequence alignment of OPA1 protein sequences of *Homo sapiens* (NP_056375.2), *Mus musculus* (NP_598513.1), and *Rattus norvegicus* (NP_598269.3). White letters on red background: conserved residues; black letters: different residues; red letters: residues with similar physicochemical properties. Green digits denote disulfide bridges. OPA1 structure (PDB: 8EF7)^31^ was used to indicate secondary structures. Alpha helix: α; 3_10_-helix: η; β-strand: black arrow; strict β-turns: TT. Clustal Omega v1.2.4^44^ was used for the alignment, and ESpript 3.0^45^ server was used for generating the image. **B)** Table showing the organism where the representative lysine residue was found to be acetylated.

**Supplementary figure 2: Analysis of s-OPA1 model reveal hydrogen bonding between lysine and interacting residues. A)** s-OPA1 dimer colored according to domain model in Fig 1. Chain B is colored in lighter hues. The PDB id for apostate (left) is 8EF7 and GDP-ALF_x_ bound state (right) is 8EEW^31^. **B and C)** Hydrogen bonding if any between lysine and interacting residue shown in red for chain A (top) and chain B (bottom) for s-OPA1 dimer. **B)** apostate (PDB: 8EF7). **C)** GDP-ALF_x_ (PDB: 8EEW). Residues are displayed with sticks and heteroatom colored: Hydrogen colored white, nitrogen colored blue, Oxygen colored red and carbon colored according to the backbone.

**Supplementary figure 3: SDS PAGE analysis of sedimentation for s-OPA1 and mutants.** Triplicates of s-OPA1 and mutants were sedimented with liposomes (**A**) and without liposomes (**B**) were analyzed using SDS-PAGE. Gels for Pellet fraction are at the top and supernatant at the bottom. **A)** Lanes are numbered: 1, WT(protein only); 2, WT; 3, K328Q; 4, K328R; 5, K342Q; 6, K342R; 7, K579Q; 8, K579R; 9, K663Q; 10, K663R; 11, K772Q; 12, K772R. **B)** Lanes are numbered: 1, WT; 2, K328Q; 3, K328R; 4, K342Q; 5, K342R; 6, K579Q; 7, K579R; 8, K663Q; 9, K663R; 10, K772Q; 11, K772R.

**Supplementary figure 4: Cardiolipin enriched liposomes visualized with EM.** Negative staining electron micrographs of cardiolipin containing liposomes in the absence (left) and presence of GMPPNP (right). Addition of GMPPNP causes liposome aggregation. Scale bars are 500 nm.

**Supplementary figure 5: Characterization of s-OPA1 GTP hydrolysis.** Total free phosphate measured after 60 minutes for Dynamin related protein 1 isoform 2 (DRP1), OPA1 WT and mutants using malachite green assay. The bar graph shows mean ± s.e.m of triplicates in the presence and absence of cardiolipin containing liposomes.
